# Genetic assessment of consecutively recruited dystonia cases from a single center

**DOI:** 10.1007/s10048-026-00922-2

**Published:** 2026-07-22

**Authors:** Burcu Atasu, Javier Simón-Sánchez, Ann-Kathrin Hauser, Peter Heutink, Thomas Gasser, Ebba Lohmann

**Affiliations:** 1 German Center for Neurodegenerative diseases (DZNE)-Tübingen, Tübingen, Germany; 2 Hertie Institute for Clinical Brain Research, University of Tübingen, Tübingen, Germany; 3 Department of Neurology, Tübingen University, Hoppe-Seyler-Str.3, 72070 Tübingen, Germany

**Keywords:** Genetics, Dystonia, Case series, Exome sequencing

## Abstract

The genetics of dystonia have been extensively investigated in population-based and multicenter cohorts. In this study, we analyzed a consecutively recruited, single-center German dystonia cohort using whole-exome sequencing (*n* = 153 affected individuals [*n* = 152 index cases from 152 dystonia families], *n* = 10 unaffected family members). Pathogenic or likely pathogenic variants were identified in established (*n* = 7) and less-established (*n* = 4) dystonia-associated genes, resulting in a diagnostic yield of 7.2% (11/152); all variants were heterozygous. Consistent with previous European studies, earlier age at onset (≤ 40 years; p value = 0.0218) was significantly associated with increased diagnostic yield and demonstrated acceptable predictive value (AUC = 0.76). This study (1) underscores the importance of both shared features—such as clinical characteristics associated with diagnostic yield—and distinct genetic features, including a relatively lower diagnostic rate and differences in the composition of implicated genes across cohort designs, and (2) suggests that exome-based testing may identify clinically relevant variants beyond the classic dystonia genes in selected patients undergoing diagnostic evaluation.

**Supplementary Information:**

The online version contains supplementary material available at 10.1007/s10048-026-00922-2.

## Introduction

Dystonia is one of the most common movement disorders, accounting for approximately 20% of patients seen in movement disorders clinics [1, 2]. It is characterized by sustained or intermittent muscle contractions that cause abnormal, often repetitive movements, postures, or both [3].

Dystonia is a genetically and clinically highly heterogeneous disorder with a complex underlying etiology. With the advent of next-generation sequencing, large-scale studies have identified more than 400 established or candidate dystonia-associated genes across cohorts [4] with varying clinical compositions, yielding diagnostic rates ranging from 8.1% to 36% [5–8]. Despite rapid gene discovery, these advances have not substantially altered diagnostic strategies, likely due to the marked clinical heterogeneity observed across populations.

In this study, we analyzed a prospectively recruited single-center German dystonia cohort with prominent dystonic features using whole-exome sequencing (WES) (*n* = 153 affected individuals [*n* = 152 index cases], *n* = 10 unaffected family members). We assessed the frequency of pathogenic variants in dystonia-associated genes and compared the clinical characteristics of this cohort with those reported in recently published studies.

## Materials and methods

### Patient’s characteristics and clinical approach

A total number of 153 affected individuals with dystonia from 152 dystonia families (including five trio) with German origin were prospectively recruited into the study at the Department for Neurodegenerative Diseases, Center for Neurology, Tübingen, Germany, between the years of 2015–2018. The mode of inheritance in all analyzed families was consistent with either autosomal dominant or sporadic patterns. All the affected members (*n* = 153) and their available unaffected family members (*n* = 10) underwent detailed clinical examination. 41 of 152 (27%) families had a positive family history. The median age at onset (AAO) is 40, ranging from 1 to 81. Radiological and laboratory findings were obtained from all the recruited individuals when it was deemed necessary. All the clinical characteristics are summarized in Table 1. The written consent was obtained from all the subjects recruited, and the study was approved by the Ethics Committee of Tübingen.

**Table 1:** Summary of the clinical characteristics of the dystonia cohort

	Affected cases with Pathogenic of Likely Pathogenic Variants in the Dystonia-Associated Genes (*n* = 11)	Affected cases with VUS (*n* = 54)	Total Affected Families (*n* = 152)
Clinical CharacteristicsAge at onset			
Infancy (birth to 2 years)	2 (18.2%)	0	5 (3.3%)
Childhood (3–12 years)	1 (9%)	4 (7.4%)	9 (5.9%)
Adolescence (13–20 years)	3 (27.3%)	6 (11.1%)	15 (9.9%)
Early adulthood (21–40 years)	3 (27.3%)	13 (24.1%)	49 (32.2%)
Late adulthood (> 40 years)	2 (18.2%)	31 (57.4%)	74 (48.7%)
***Body distribution***			
Focal	7 (63.6%)	43 (79.6%)	111 (73%)
Segmental/Multifocal	0	3 (5.6%)	17 (11.2%)
Generalised	4 (36.4%)	8 (14.8%)	24 (15.8%)
***Associated features***			
Isolated dystonia	9 (81.8%)	32 (59.2%)	92 (60.5%)
Combined dystonia	2 (18.2%)	22 (40.8%)	60 (39.5%)
***Additional Clinical Characteristics***			
Ataxia	1 (9%)	0	1 (0.6%)
Choreoathetosis Epilepsy and seizures Cerebellar disease	1 (9%) 1 (9%) 1 (9%)	0 2 (3.7%) 1 (1.8%)	1 (0.6%) 2 (1.3%) 2 (1.3%)
Developmental delay	1 (9%)	2 (3.7%)	8 (5.2%)
Parkinsons disease	0	5 (9.3%)	10 (6.6%)
Peripheral Neuropathy	0	0	1 (0.6%)
Restless Legs Syndrome	0	0	2 (1.3%)
Spastic Paraparesis Psychiatric disorder	0 0	0 4 (7.4%)	1 (0.6%) 2 (1.3%)
***MRI findings***			
Hydrocephalus	0	0	1 (0.6%)
Cerebellar atrophy	1 (9%)	2 (3.7%)	9 (5.9%)

### Genetic analyses

In this study, 163 whole exome sequenced samples (*n* = 153 affected cases [*n* = 152 index cases], *n* = 10 unaffected family members) were pre- and post-processed as described elsewhere [8]. Briefly, raw data were preprocessed using our in-house pipelines to generate variant call format (vcf) files and resulting vcf files were post-processed to retrieve variants of disease-associated genes listed elsewhere [8].

Following variant filtering and prioritization, all the retrieved dystonia-associated variants were classified using InterVar, VarSome, Franklin [9–11] based on published standards and guidelines for the clinical interpretation of sequence variants by the American College of Medical Genetics and Genomics (ACMG) and the Association for Molecular Pathology (AMP) [12]. Heterozygous variants were retained only if absent from control populations in the GnomAD control database (GnomAD v3.1.2 Controls) [13]. Loss-of-function variants were filtered out when located in genes tolerant to such variation, as defined by a pLI score of 0. Missense variants were evaluated using AlphaMissense [14] and, and splicing variants were assessed using Splice AI [15].

In parallel, copy number variations (CNVs) were investigated using XHMM (eXome-Hidden Markov Model) as described elsewhere [8].

The study work flow, and variant filtering-prioritisation steps were illustrated in the supplementary material S1.

All pathogenic and likely pathogenic variants were validated by Sanger sequencing, with segregation analysis performed when DNA samples were available.

### Statistical analyses

Associations between clinical characteristics and genetic findings were assessed using statistical analyses. Fisher’s exact test was performed in Python to evaluate associations between clinical subgroups (isolated, combined, generalised, segmental, and focal dystonia; dystonia with additional features; and positive family history) and genetic findings. The Mann–Whitney U test was used to assess the association between AAO and genetic findings. Analysis of variance (ANOVA) was conducted to examine the association between variant type and AAO. Receiver operating characteristic (ROC) analysis was performed using the pROC package to evaluate the predictive value of genetic findings for diagnostic yield across clinical subgroups. The Mann–Whitney U test, ANOVA, and ROC analyses were conducted in R (version 4.5.1). Data visualization was performed using the ggplot2 package.

## Results

### Overview of the genetic findings

**Fig. 1:**
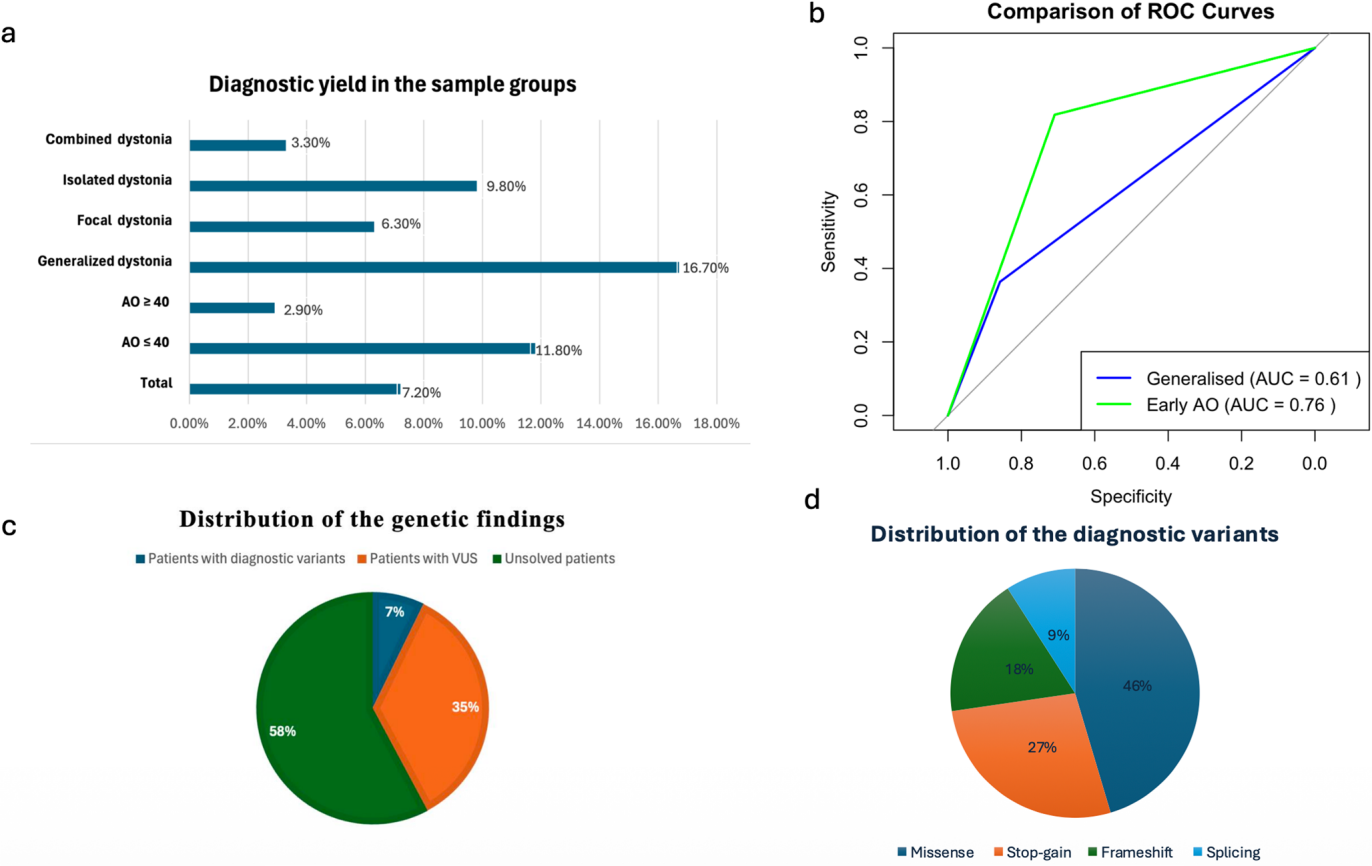
(**a**) The chart illustrates the diagnostic yields by patient groups based on the clinical characteristics. (**b**) ROC curve plot illustrates the predictive value of generalised dystonia prevalence and early AAO (≤ 40 years) for the diagnostic value in our cohort. A ROC curve plots the true positive rate (sensitivity) on the y-axis against the false positive rate (1 − specificity) on the x-axis across a range of decision thresholds. (**c**) The chart illustrates the distribution of samples according to genetic findings. (**d**) The chart illustrates the distribution of variant types

Following the application of stringent variant filtering and prioritization criteria, WES identified (1) pathogenic or likely pathogenic variants in established dystonia-associated genes in seven families (2.8%), as well as in genes associated with a broad spectrum of clinical phenotypes in which dystonia may represent a less prominent feature in four families (1.6%) (Table 2, Supplementary material S2), and (2) variant of unknown significance (VUS) in 54 families (35%) (Fig. 1c, Supplementary material S3).

**Table 2:** The characteristics of the identified variants in the dystonia associated genes

Gene symbol		Variant/Type	GnomAD v3.1.2 Controls MAF^a^/Previous Report		Pathogenicity based on InterVar, VarSome, Franklin^b^	Pathogenecity Evidence	Zygosity/ Inheritance	Alpha missense score^c^/Splice AI Predictions^d^	Genotype-phenotype Correlation^f^	MDS Gene/Clinical characteristics	Patient ID (*N* = 11) ^d^
	Findings in the Established Dystonia Genes										
SGCE		NM_003919.3:c.193G > T:p.Glu65Ter/Stop-gained	0/NA		P, P, P	PS4,PVS1, PM2, PM6	H/Sporadic	NA/NA	Consistent	DYT-SGCE	28382
KMT2B		NM_014727.3: c.4847 C > T:p.Ala1616Val/Missense	0/NA		LP, VUS, LP	PS4, PM2, PP2	H/NA	Pathogenic/NA	Consistent	DYT- KMT2B	10369
VPS16		NM_022575.4: c.1389 C > G:p.Tyr463Ter/Stop-gained	0/NA		LP, P, P	PS4, PVS1, PM2	H/AD	NA/NA	Consistent	DYT-VPS16	3137
GNAL		NM_182978.4: c.964 C > T:p.Arg322Ter/Stop-gained	0/ClinVar Pathogenic		P, P, P	PS4, PVS1, PM2	H/NA	NA/NA	Consistent	DYT-GNAL	10398
ANO3		NM_031418.4:c.2267T > C: p.Leu756Ser/Missense	0/NA		LP, VUS, VUS	PM2, PP3	H/Sporadic	Pathogenic/NA	Consistent	DYT-ANO3	21103
THAP1		NM_018105.3: c.270_273del: p.Glu91IlefsTer28/Frameshift	0/ClinVar Pathogenic		LP, P, P	PS4, PVS1, PM2	H/AD	NA/NA	Consistent	DYT-THAP1	21339
EIF2AK2		NM_001135651.3:c.−16-1G > A/Splicing	0/NA		LP, B, LP	PP4, PM2, PVS1	H/Sporadic	NA/Acceptor loss score 1	Consistent	DYT- EIF2AK2	29928
	Findings in the Movement Disorders Genes Associated with Dystonia										
TET3		NM_001287491.2: c.3831dup p.Lys1278GlnfsTer8/Frameshift.	0/NA	LP, LP, LP		PVS1,PM2,PM6	H/Sporadic	NA/NA	Partially consistent [18]	NA/Beck–Fahrner syndrome (MIM 618798)	28963
NKX2-1		NM_001079668.3:c.872 C > T:p.Pro291Leu/Missense	0/NA	P, P, P		PS4, PM2, PM5, PP3, PM6	H/Sporadic	Pathogenic/NA	Consistent	CHOR (MIM 118700)	28259
CACNA1G		NM_018896.5 :c.4507 C > A:p.Gln1503Lys/Missense	0/NA	LP, B, LP		PP4, PM2, PP3, PP2	H/AD	Ambiguous/NA	Partially consistent [19-21]	Spinocerebellar Ataxia type 42 (SCA42; MIM 616795)	21307
VPS4A		NM_013245.3: c.1234G > A:p.Ala412Thr/Missense	0/NA	LP, B, LP		PP4, PM2	H/NA	Benign/NA	Partially consistent [22-24]	CIMDAG syndrome (MIM 619273)	15,242

a: GnomAD based MAF in controls b: ACMG/AMP based pathogenicity classification. c: AlphaMissense outputs a probability (0–1) that a missense variant is damaging. d: Splice AI outputs Δ score (0–1) for acceptor/donor gain. d: All the cases with pathogenic or likely pathogenic variants. H: heterozygous. AD: Autosomal dominant

Within the diagnostic group, the most common clinical features were adolescent (27.3%) or early adulthood onset (27.3%) dystonia, focal dystonia (63.6%), and isolated dystonia (81.8%). Additional features were present in only 9% of patients carrying pathogenic or likely pathogenic variants (Table 1). Among clinical characteristics, only generalised dystonia (Fisher’s exact test, p value = 0.07) approached significance, while earlier age at onset (≤ 40 years; Mann–Whitney U test, p value = 0.0218) was significantly associated with a genetic diagnosis. Other subgroups, including cases with VUS, showed no statistical association. ROC analysis indicated that generalised dystonia (16.7%; AUC = 0.61) provided limited discriminative value, whereas early onset (≤ 40 years; 11.8%; AUC = 0.76) offered acceptable predictive value for diagnostic yield (Fig. 1a and b). Given the small sample size results should be interpreted with caution.

Pedigrees of the available families with diagnostic variants were illustrated in the supplementary material S4.

### Genetic findings

Our analysis revealed 7 pathogenic or likely pathogenic variants in seven established dystonia genes: *SGCE* (*n* = 1*)*, *KMT2B* (*n* = 1), *VPS16* (*n* = 1*)*, *GNAL* (*n* = 1), *ANO3* (*n* = 1), *THAP1* (*n* = 1), and *EIF2AK2* (*n* = 1). Additionally, four pathogenic or likely pathogenic variants were detected in genes not directly associated with dystonia- *TET3* (*n* = 1), *NKX2-1* (*n* = 1), *CACNA1G* (*n* = 1), and *VPS4A* (*n* = 1)*-* which are linked to a broader spectrum of clinical phenotypes, with dystonia often representing a less prominent feature.

Among the identified variants, the majority were missense (*n* = 5), followed by stop-gain (*n* = 3), frameshift (*n* = 2), and splicing variants (*n* = 1) (Fig. 1d; Table 2).

Except for two variants (*GNAL*, p.Arg322Ter; *THAP1*, p.Glu91IlefsTer28), all other variants in this group were novel, with one (*NKX2-1*, p.Pro291Leu) occurring *de novo*.

Pathogenic or likely pathogenic variants identified in less-established dystonia-associated genes in our cohort were primarily linked to other movement disorders. Specifically, *TET3*, *NKX2-1*, *CACNA1G*, and *VPS4A* are associated with Beck–Fahrner syndrome (MIM 618798), benign hereditary chorea (MIM 118700), spinocerebellar ataxia type 42 (SCA42; MIM 616795), and CIMDAG syndrome (MIM 619273), respectively.

Only one copy number variation (*TUBB4A*, NM_006087.4:c.286_406dup) was identified; however, it was excluded from the list because it has been reported in 25 heterozygous and 3 homozygous individuals in gnomAD.

All pathogenic and likely pathogenic variants have been deposited in ClinVar (SUB15939327, SUB15939298).

## Discussion

Dystonia is a clinically and genetically highly heterogeneous group of disorders with complex underlying pathophysiology. Clinically, dystonia might be an initial manifestation of a complex multi-system neurological disorder or only a sole clinical feature [16]. This clinical complexity of dystonia makes genetic studies more challenging than usual.

Although dystonia has been extensively investigated genetically—with ongoing gene discovery, replication studies, and genome-wide analyses including structural and non-coding variants—the majority of cases with a suspected genetic etiology remain undiagnosed [5]. Several factors may hinder genetic diagnosis, including the limited availability of informative families with well-defined clinical phenotypes, reduced gene penetrance, and technical limitations of current screening approaches. To address these challenges, large-scale, systematic, multi-population genome-wide studies have been conducted, yielding diagnostic rates ranging from 8.1% to 36%, largely depending on the composition of the study population.

In this study, we assessed the diagnostic yield of a prospectively recruited, single-center German dystonia cohort comprising 152 families with dystonic features. Our analysis revealed that: (1) the overall diagnostic rate was relatively low (7.2%) compared to the study by Zech et al. (21.7%) [17] and similar to the study by Thomsen et al. (8%) [7] (Supplementary material S5), (2) consistent with prior reports, generalised dystonia (*p* = 0.07) and early age at onset (≤ 40 years; *p* = 0.02) were the main clinical features associated with a genetic diagnosis, and (3) in comparison to the study by Thomsen et al., disease-causing variants were distributed similarly between established dystonia genes (Thomsen et al., 74%; this study, 63.6%) and less-established dystonia-associated genes (Thomsen et al., 26%; this study, 36.4%).

In the previous study by Thomsen et al., in addition to generalised dystonia and earlier age at onset, a positive family history and the presence of additional clinical features were also associated with diagnostic yield. Although several similarities exist between that study and ours, these differences may reflect variations in recruitment strategies. Notably, despite a relatively high prevalence of focal dystonia in both cohorts (this study: 111/152 [73%]; Thomsen et al.: 1045/1924 [54.3%]), no association with diagnostic yield was observed. This finding may indicate a weaker contribution of rare variants to the pathogenesis of focal dystonia.

In contrast to InterVar, four variants in *CACNA1G*, *ANO3*, *VPS4*, and *EIF2AK2* were classified as benign or VUS by the confirmatory prediction tools VarSome and Franklin. Nevertheless, all were included in the diagnostic group because (1) the associated clinical phenotypes were consistent with reported disease presentations, and (2) the variants were extremely rare in population databases.

In addition to the pathogenic variants, our analysis revealed VUS variants in 54 families (35%). The stringent filtering criteria applied in our study, along with limited availability of detailed clinical information from family members, may have contributed to the high number of VUS identified. Nevertheless, no differences in clinical characteristics were observed between unsolved cases and those carrying VUS.

In this study, *SGCE*, *NKX2-1*, *KMT2B*, *VPS16*, *GNAL*, *ANO3*, *THAP1*, and *EIF2AK2* overlapped with genes previously reported by Thomsen et al. and Zech et al., whereas *TET3*,* CACNA1G*, and *VPS4A* were uniquely identified in our cohort, highlighting genetic contributors that may be encountered in clinical practice within these populations (Supplementary Material S6). Among these, the less-established dystonia-associated genes (*TET3*,* NKX2-1*,* CACNA1G*, and *VPS4A;*for a more detailed clinical presentation see Supplementary material S7) are primarily linked to neurodevelopmental disorders, followed by chorea and ataxia, which are thought to share convergent disease mechanisms with dystonia. Notably, mostly all of those genes (*TET3*,*CACNA1G*, and *VPS4A*) except for *NKX2-1*were not identified in previous genome-wide analyses of dystonia cohorts.

The clinical presentations of cases with variants in *TET3*, *CACNA1G*, and *VPS4A* were only partially consistent, as dystonia represented the predominant but not exclusive phenotype (Supplementary material S7). While these genes have been implicated in dystonia, they are also associated with a broader spectrum of neurological disorders, in which dystonia may emerge as a prominent feature [18–24]. We therefore included these cases in the diagnostic group, acknowledging the expanding phenotypic spectrum and the potential for pleiotropic effects.

Our findings (1) suggest that even relatively small, consecutively recruited single-center cohorts can identify similar clinical determinants underlying the genetic basis of dystonia; (2) highlight that exome-based testing may identify clinically relevant variants beyond the classic dystonia genes in selected patients undergoing diagnostic evaluation due to the broad genetic heterogeneity of dystonia; and (3) indicate that certain features, including the distribution of identified genes, may vary depending on cohort characteristics and study design.

## Supplementary Information

Below is the link to the electronic supplementary material.

Supplementary Material 1 (PDF 913 KB)

## Data Availability

The datasets generated during and/or analysed during the current study are available from the corresponding author on reasonable request.
