# Supplementary material for: Genetic assessment of consecutively recruited dystonia cases from a single center
Source: Neurogenetics. 2026 Jul 22;27(1):51. doi: 10.1007/s10048-026-00922-2 (PMC13391772; doi:10.1007/s10048-026-00922-2)
Supplement: Supplementary file 1 — Supplementary Material 1 (PDF 913 KB) [file 10048_2026_922_MOESM1_ESM.pdf]

Supplementary Material

S1 STUDY WORKFLOW 2

S2 CLINICAL CHARACTERISTICS OF THE PATIENTS WITH THE PATHOGENIC OR LIKELY PATHOGENIC VARIANTS 3

S3 CLINICAL CHARACTERISTICS OF THE PATIENTS WITH VUS 3

S4 PEDIGREES OF THE FAMILIES WITH DIAGNOSTIC VARIANTS 5

S5 DIAGNOSTIC RATES AND STUDY POPULATIONS IN RECENT STUDIES 6

S6 LIST OF THE COMMON AND DISTINCT GENES IMPLICATED IN DIFFERENT DIAGNOSTIC GROUPS BY STUDY 7

S7 DETAILED CLINICAL CHARACTERISTICS CORRESPONDING TO THE SAMPLES WITH *TET3*, *NKX2-1*, *CACNA1G*, *VPS4A* 9

S1 Study Workflow

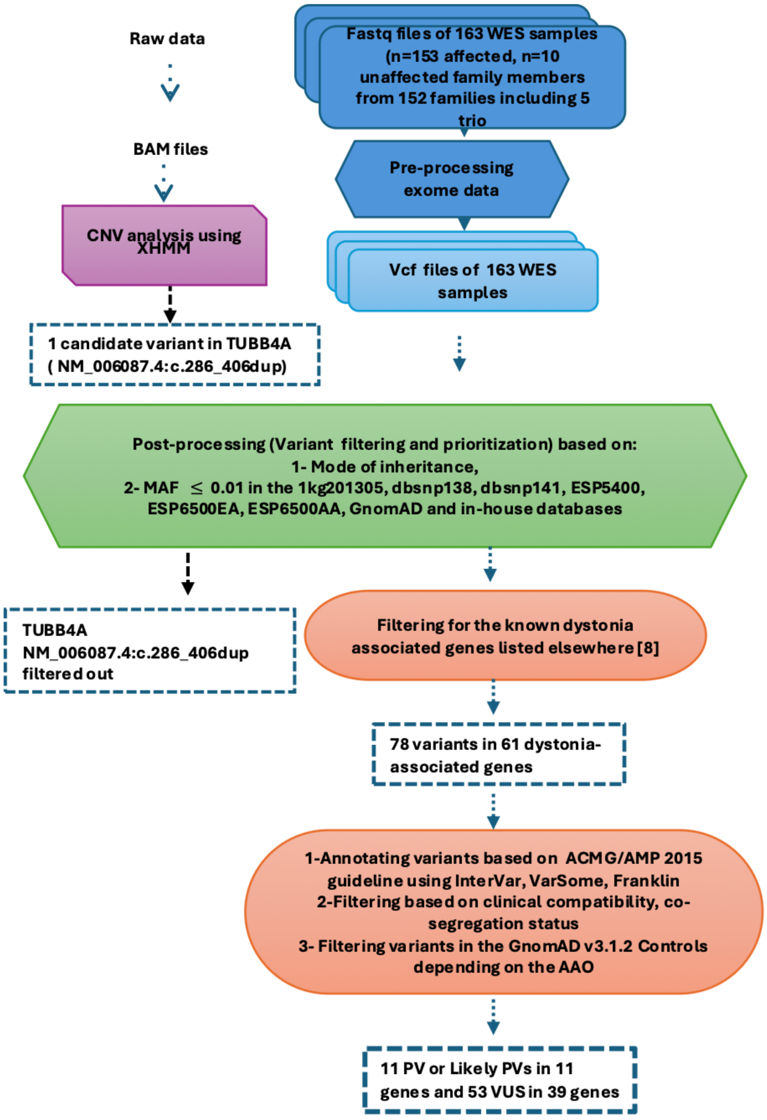

☆

Systematic illustration of the workflow. The figure illustrates the systematic screening approach conducted to analyze the 152 dystonia families.

S2 Clinical Characteristics of the patients with the pathogenic or likely pathogenic variants

Patient ID	Clinical Characteristics
28382	Myoclonus-dystonia. AO: 37. No other neurological symptoms.
10369	Generalized dystonia. AO:20. No family history.
3137	Generalized dystonia. AO:6. Positive family history.
10398	Focal dystonia. Subtype: Cervical dystonia. AO:17. No family history.
21103	Focal dystonia. Subtype: Cervical dystonia. AO:20. No family history.
21339	Focal dystonia. AO:40. Positive family history.
29928	Cervical dystonia, tremor. AO:48.
28963	Adult-onset combined segmental dystonia with dystonic tremor and marked levodopa responsiveness. AO:32. No family history.
28259	Combined generalized dystonia with myoclonus, chorea, dystonic tremor, developmental delay, and oculomotor abnormalities, likely due to an underlying genetic neurodevelopmental/neurodegenerative disorder. AO:1. No family history.
21307	Subtype: Cervical dystonia, Tremor. AO: 70. MRI: Steep cervical spine with slight retro curves in the middle and lower cervical thirds. Positive family history.
15242	Generalized dystonia. AO: 1. No family history.

S3 Clinical Characteristics of the patients with VUS

ID	Body Distribution of Dystonia	Type of Dystonia	Variant
13479	Generalized	Isolated	KMT2B:NM_014727:c.4463G>A:p.R1488Q
14406	Focal	Isolated	COL4A1:NM_001303110:c.7C>A:p.P3T
17709	Segmental	Isolated	PANK2:NM_153638:c.131G>T:p.R44L
25185	Generalized	Isolated	PI4KA:NM_058004:c.2665A>G:p.M889V
27678	Focal	Combined	FUS:NM_004960:c.1240G>A:p.G414S:
27765	Generalized	Combined	ATP7B:NM_001005918:c.1621G>A:p.E541K
28122	Focal	Combined	PDGFB:NM_002608:c.115C>A:p.R39S
28338	Segmental	Isolated	HECW2:NM_020760:c.3040A>G:p.I1014V
29711	Focal	Isolated	SPR:NM_003124:c.472C>G:p.L158V
29735	Focal	Combined	DHX30:NM_138615:c.87G>A:p.M29I
29848	Focal	Combined	AFG3L2:NM_006796:c.790A>G:p.I264V
29988	Focal	Combined	TH:NM_199292:c.67G>A:p.A23T
30303	Focal	Isolated	GNAL:NM_182978:c.265G>A:p.A89T
30617	Focal	Combined	TRPM7:NM_017672:c.4084C>T:p.P1362S
30793	Focal	Isolated	CACNA1A:NM_023035:c.7091G>A:p.G2362D
30957	Focal	Combined	LRRK2:NM_198578:c.3683G>C:p.S1228T

31171	Focal	Isolated	ADCY5:NM_183357:c.2818G>A:p.E940K
31191	Focal	Isolated	CACNA1B:NM_001243812:c.4738C>T:p.R1580C
31291	Focal	Combined	PPP2R2B:NM_181677:c.647A>G:p.N216S
31302	Focal	Isolated	TET3:NM_001287491:c.55G>A:p.D19N
31378	Focal	Combined	SGCE:NM_001099401:c.391T>C:p.I131V
31527	Focal	Combined	NKX2-1:NM_001079668:c.1187T>C:p.L396P
31568	Focal	Combined	VPS4A:NM_013245:c.1226G>A:p.R409Q
31616	Generalized	Isolated	RELN:NM_005045:c.139G>A:p.E47K
31742	Segmental	Combined	ANO3:NM_001313727:c.2374G>A:p.V792I
31813	Focal	Combined	HIVEP2:NM_006734:c.7163A>G:p.D2388G
32070	Focal	Isolated	IFIH1:NM_022168:c.A802delAGAAGGAAGTGTCAGC-:p.S268delEGSVS
32175	Focal	Isolated	PLA2G6:NM_001199562:c.101C>T:p.S34L
32294	Focal	Isolated	SCN8A:NM_001177984:c.4612T>C:p.Y1538H
32498	Generalized	Isolated	GCH1:NM_001024024:c.328C>G:p.Q110E
32602	Focal	Isolated	PPP2R2B:NM_181677:c.881G>A:p.R294H
32714	Focal	Isolated	KMT2B:NM_014727:c.3895G>T:p.A1299S
32719	Focal	Isolated	IRF2BPL:NM_024496:c.1220C>T:p.A407V
32743	Generalized	Isolated	THAP1:NM_018105:c.238A>G:p.I80V
32758	Focal	Isolated	PRRT2:NM_001256443:c.224C>T:p.P75L
33086	Focal	Combined	CACNA1B:NM_000718:c.6965C>T:p.S2322L
33427	Generalized	Combined	PRKRA:NM_001316362:c.365G>C:p.S122T
33487	Generalized	Isolated	TUBB4A exon4 duplication
33499	Focal	Isolated	CBS:NM_000071:c.1643G>A:p.R548Q
33583	Focal	Combined	SCN8A:NM_001177984:c.3685C>T:p.R1229C
33607	Focal	Isolated	CACNA1A:NM_000068:c.3052G>A:p.E1018K:
33698	Focal	Isolated	HIVEP2:NM_006734:c.944T>G:p.L315W
33845	Focal	Combined	SGCE:NM_001099400:c.1280G>A:p.R427H
34491	Focal	Isolated	LRRK2:NM_198578:c.2248G>A:p.E750K
35053	Focal	Isolated	KMT2B:NM_014727:c.G6754ins+CCCCC
35126	Focal	Isolated	ADCY5:NM_183357:c.1024C>G:p.L342V
35271	Focal	Isolated	PLA2G6:NM_003560:c.1267G>A:p.A423T
35446	Focal	Isolated	KMT2B:NM_014727:c.1391C>T:p.T464M
35473	Focal	Combined	PANK2:NM_153638:c.796G>A:p.V266M
35496	Focal	Isolated	ALDH18A1:NM_002860:c.211A>G:p.K71E
35695	Focal	Isolated	PRKAR1B:NM_001164759:c.949C>T:p.R317C
36131	Focal	Combined	CACNA1A:NM_000068:c.3187C>T:p.P1063S
36331	Generalized	Combined	SPTBN1:NM_178313:c.2210A>G:p.Q737R
36606	Generalized	Combined	TH:NM_199292:c.691C>T:p.R231C

S4 Pedigrees of the Families with Diagnostic Variants

28382-Sporadic

SGCE NM_001099401.2 p.(Glu65Ter)

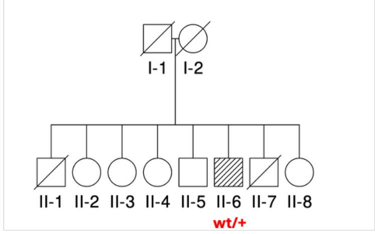

3137-AD

VPS16 NM_022575.4 p.Tyr463Ter

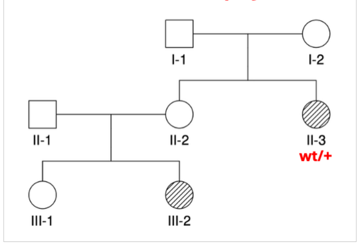

21103-Sporadic

ANO3 NM_001313726.2 p.(Leu817Ser)

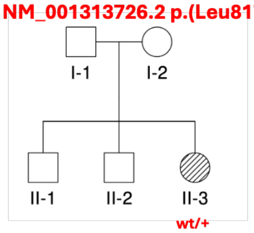

21339-AD

THAP1 NM_018105.3 p.(Glu91IlefsTer28)

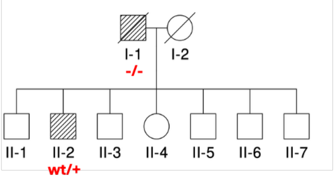

29928-Sporadic

EIF2AK2 NC_000002.11c.-16-1G>A

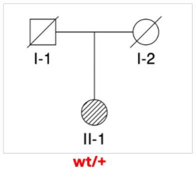

28963-Sporadic

TET3 NM_001287491.2 p.(Lys1278GlnfsTer8)

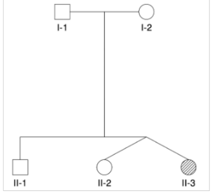

28259-Sporadic

NKX2-1 NM_001079668.3 p.(Pro291Leu)

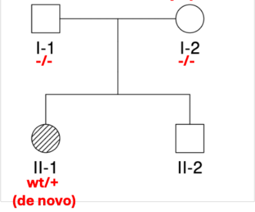

21307-AD

CACNA1G NM_001256326.2 p.(Gln1503Lys)

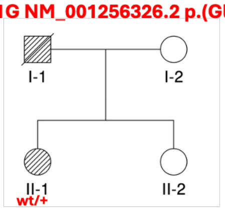

All the avaible pedigrees in the diagnostic group is illustrated. AD: Autosomal dominant. Wt: Wild type.

S5 Diagnostic rates and study populations in recent studies

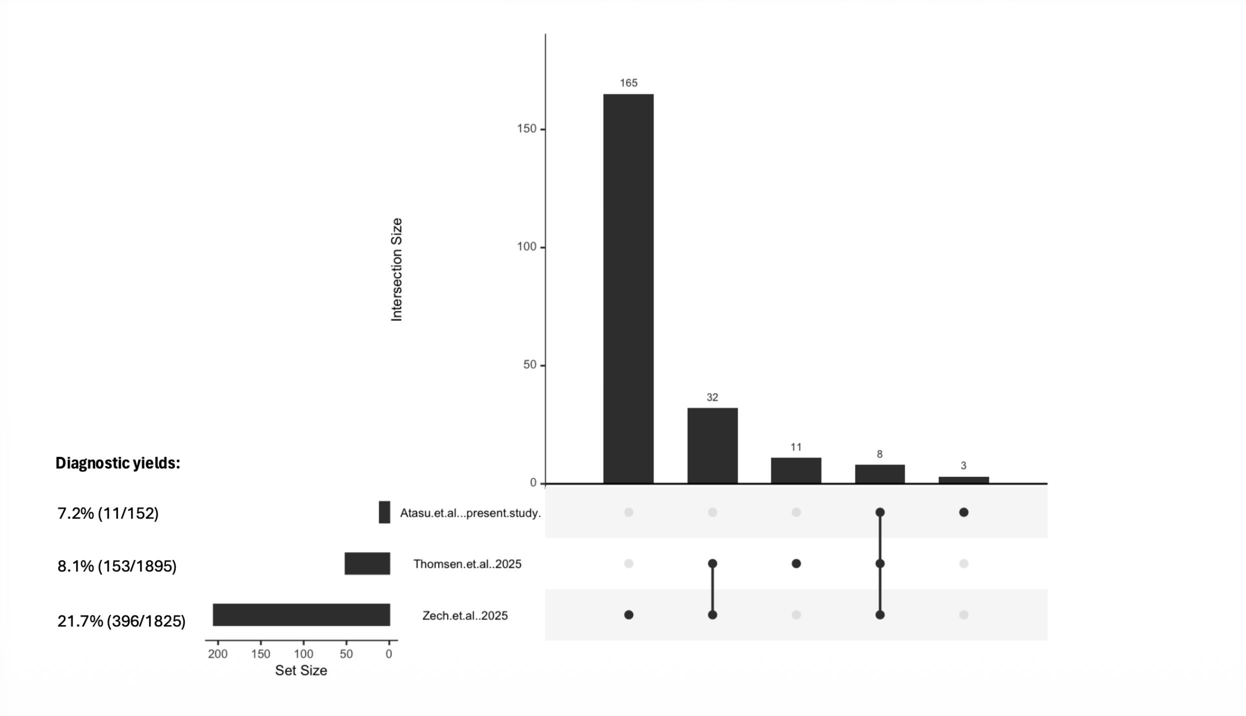

The figure illustrates a comparison of diagnostic yields, study sizes, and the convergent and divergent gene pools (genes are described in Supplementary Table S5) across three recent studies: Zech et al. (2025), Thomsen et al. (2025), and the present study.

S6 List of the Common and Distinct Genes implicated in different diagnostic groups by study

Study	Gene List	Number of Genes
Thomsen et al., 2025	ACTB, ADCY5, AFG3L2, ANO3, ATL1, ATP1A3, C19orf12, CACNA1A, CHD6, CHMP2B, COL4A1, CTNNB1, EIF2AK2, EIF4A2, FTL, GCH1, GNAL, GNAO1, GNB1, GRN, IFIH1, IRF2BPL, KCNJ10, KCNMA1, KCNN2, KCNQ2, KCTD7, KIF1A, KLC1, KMT2B, MECP2, NKX2-1, NR4A2, PDE10A, PRKCG, PSEN1, SCN8A, SETX, SGCE, SLC2A1, SLC6A1, SPAST, SPR, TBP, TCF20, THAP1, TOR1A, TUBB4A, TWNK, VPS16, ZMYND11	51
Zech et al., 2025	AARS1, ACTB, ADAR, ADCY5, AFG3L2, ALS2, ANK2, ANO3, AOPEP, ARHGEF9, ARSA, ASXL3, ATL1, ATM, ATP1A3, ATP2B2, ATP5F1A, ATP5F1B, ATP5MC3, ATP7B, ATP8A2, AUTS2, BCL11B, BRAF, BRPF1, C19orf12, CACNA1A, CACNA1E, CAMK4, CAMTA1, CASK, CD40LG, CHD3, CHD4, CHD8, CNTNAP1, COQ8A, CP, CSDE1, CTNNB1, CUL3, CUX1, CWF19L1, DCAF17, DDC, DHCR24, DHDDS, DLG4, DLL1, DNAJC6, DNM1L, DNMT1, EBF3, ECHS1, EEF1A2, EFTUD2, EIF2AK2, EIF4A2, ERCC4, ERCC8, FA2H, FBXO31, FGF14, FITM2, FOXG1, FOXP2, FRMD5, FRYL, FTL, GABBR2, GABRA1, GAD1, GCH1, GJA1, GJC2, GNAL, GNAO1, GNB1, GRIA2, GRIA3, GRID2, GRIN1, GRIN2A, HECW2, HEXA, HIBCH, IFIH1, IMPDH2, INTS11, IRF2BPL, KCNA2, KCNB1, KCNJ10, KCNMA1, KCTD17, KIF1A, KIF5A, KMT2B, LIG4, LRRK2, MAG, MATR3, MECP2, MECR, MED23, MICU1, MMAA, MORC2, MRE11, MSL3, NAA15, NARS2, NAV3, NEFL, NFIX, NGLY1, NKX2-1, NPC1, NR4A2, NUP54, OPA1, PAK1, PANK2, PARK7, PCDH12, PDE10A, PDHA1, PINK1, PLA2G6, PNKD, PNPLA6, POGZ, POLG, POLR1A, POLR3A, PPP2R5D, PPT1, PRKCG, PRKN, PRRT2, PSEN1, PTS, PURA, RALA, RARB, RERE, RHOBTB2, SATB1, SCN2A, SCO2, SCP2, SERAC1, SETX, SGCE, SHANK3, SHQ1, SLC16A2, SLC19A3, SLC20A2, SLC2A1, SLC6A1, SLC6A3, SLC9A6, SNAP25, SNX14, SON, SOX2, SOX6, SPAST, SPG11, SPG7, SPR, SPTBN1, SRRM2, SUCLG1, SUOX, SYNE1, TBC1D24, TBCD, TBX1, TCF20, TECPR2, TFE3, TH, THAP1, TMEM240, TOR1A, TTPA, TUBB4A, UBE3A, UBTF, VLDLR, VPS16, WAC, WARS2, WASHC5, WDR45, WDR73, WFS1, YY1, ZC4H2, ZEB2, ZMYND11, ZNF142, ZNF335	205
Atasu et al. (present study)	TET3, SGCE, NKX2-1, CACNA1G, KMT2B, VPS16, GNAL, ANO3, THAP1, VPS4A, EIF2AK2	11
Common genes in all three studies	SGCE, NKX2-1, KMT2B, VPS16, GNAL, ANO3, THAP1, EIF2AK2	8

Common genes in Thomsen et al. (2025) and Zech et al. (2025)	ACTB, ADCY5, AFG3L2, ANO3, CACNA1A, CTNNB1, EIF2AK2, FTL, GCH1, GNAL, GNB1, IRF2BPL, KCNJ10, KCNMA1, KIF1A, KMT2B, NKX2-1, NR4A2, PDE10A, PRKCG, PSEN1, SETX, SGCE, SLC2A1, SPAST, SPR, TCF20, THAP1, TOR1A, VPS16, ZMYND11	31

S7 Detailed Clinical Characteristics Corresponding to the Samples with *TET3*, *NKX2-1*, *CACNA1G*, *VPS4A*

Sample 28963 with TET3 variant:

The patient's medical history is notable for a peripheral left-sided facial nerve palsy occurring after surgical resection of a left parotid tumor in March 2012. Recovery was incomplete, and the patient subsequently developed postparalytic facial synkinesis, diagnosed in June 2013.

Since May 2014, the patient has been treated with botulinum toxin injections for facial synkinesis, which have been well tolerated and have provided satisfactory symptomatic control without adverse effects.

Toward the end of 2014, the patient developed progressive motor symptoms characterized by a cramped posture initially involving the left index finger and wrist. Over time, symptoms extended to include the left foot, where an associated tremor was also observed. Subsequently, a dystonic gait disorder emerged and progressively worsened.

A therapeutic trial of levodopa was initiated, resulting in complete initial symptom resolution. In the subsequent disease course, the patient underwent several additional surgical procedures for benign tumors. Thereafter, the clinical response to levodopa became less consistent. While the patient continues to report improvement in gait following levodopa administration, treatment is limited by the development of pronounced dysarthrophonia at higher doses.

Past medical history is additionally notable for iron deficiency anemia of unclear etiology.

The family history is remarkable for movement disorders: the older daughter is affected by writer's cramp, and the younger daughter has foot dystonia. No further information is available regarding neurological disorders in the parental generation due to long-term loss of contact.

Neurological examination revealed a clearly left-predominant dystonic syndrome. During arm extension testing, a dystonic posturing of the left hand was observed. In addition, left-sided foot dystonia was evident during gait assessment.

Mild residual left facial weakness consistent with prior facial nerve palsy was present, accompanied by a positive eyelash sign. No additional focal neurological deficits were detected.

Magnetic resonance imaging of the brain and cervical spine revealed no pathological findings.

Extensive metabolic investigations were performed in serum and cerebrospinal fluid. A phenylalanine loading test yielded normal results, arguing against a disorder of phenylalanine metabolism. Cerebrospinal fluid analysis, including amino acid profiling and neurotransmitter metabolite assessment, was largely unremarkable except for a markedly reduced tetrahydrobiopterin concentration. In addition, an elevated cerebrospinal fluid concentration of 3-O-methyldopa was detected, most likely reflecting chronic dopaminergic treatment.

Overall, the neurochemical findings remained inconclusive.

The patient has undergone multiple pharmacological treatment trials and is familiar with the full range of standard anti-dystonic medications. At present, she is maintained on low-dose levodopa, which provides partial symptomatic benefit. However, dose escalation is limited by the development of significant dysarthrophonia. Dopamine agonists were discontinued due to intolerable nausea.

Sample 28259 with NKX2-1 variant:

The patient has a history of a developmental disorder present since birth, followed by the later emergence of a hyperkinetic movement disorder. Developmental milestones were delayed, with head control achieved at approximately one year of age and independent sitting at around six months. Ambulation was significantly delayed, initially requiring assistive devices (rollator), with independent walking without support only achieved during primary school age. With ongoing therapeutic support, partial developmental gains were achieved.

Since approximately 2014, the patient has developed progressive involuntary movements characterized by myoclonic activity and dystonic posturing, predominantly involving the head. The movement disorder has shown slow but continuous progression since onset. Despite these symptoms, the patient remains independent in activities of daily living; however, involuntary movements significantly interfere with occupational and daily functioning.

Pharmacological treatment trials, including anticholinergic therapy (biperiden), tiapride, levodopa, zonisamide, tetrabenazine, and botulinum toxin injections for cervical dystonia, have failed to provide sustained or clinically meaningful benefit.

The patient completed lower secondary education, followed by vocational training as an office clerk with additional qualification in accounting. She has been employed part-time (50%) as an office clerk for the past decade. Family history is negative for neurological or movement disorders.

Neurological examination revealed a mobile cervical dystonia with left-sided torticollis and right-sided laterocollis. Cranial nerve examination was largely unremarkable apart from mildly saccadic smooth pursuit eye movements. No blepharospasm was observed.

Involuntary facial movements were present, including intermittent eyebrow elevation and deviation of the oral commissure. Examination of motor function demonstrated choreodystonic movements involving all extremities, accompanied by intermittent myoclonic jerks of lesser prominence.

Gait was narrow-based with preserved step length but exhibited a mildly stiff, dystonic-ataxic quality. No clear pyramidal weakness was identified, and there was no pronator or leg drift. Muscle bulk was generally reduced.

Oculomotor examination was limited by marked fixation instability. Mildly saccadic smooth pursuit and mild horizontal and vertical gaze paresis were observed. No square-wave jerks or pathological nystagmus were present. The vestibulo-ocular reflex was intact; saccadic metrics could not be reliably assessed.

Speech was mildly dysarthric, affecting both spontaneous speech and repetitive syllable articulation. There was no history of dysphagia.

Dystonic posturing was additionally observed in the feet and fingers. Generalized myoclonus and dystonic tremor of the upper extremities were present.

Plantar responses were difficult to assess due to cooperation and were considered questionably positive bilaterally. No definitive upper motor neuron weakness was identified.

The patient reported rare urinary incontinence (approximately twice per year) without associated urgency or other lower urinary tract symptoms. Bowel function was normal. Menarche occurred late at age 19, and menstrual cycles remain irregular.

The patient is currently undergoing evaluation for deep brain stimulation due to medically refractory generalized combined dystonia with prominent myoclonus and cervical involvement.

Sample 21307 with CACNA1G variant:

The patient presents with cervical dystonia accompanied by an intermittent horizontal head tremor of unclear origin. The initial onset of symptoms occurred at approximately 70 years of age.

Magnetic resonance imaging of the cervical spine demonstrated a steepened cervical spine alignment with mild retrocurvature involving the mid to lower cervical segments.

The family history is notable for cervical dystonia and head tremor, suggesting a possible hereditary predisposition.

Neurological examination revealed a marked left-sided laterocollis associated with mild contralateral (right-sided) torticollis. There was compensatory elevation of the right shoulder. In addition, an intermittent dystonic horizontal head tremor was observed, consistent with dystonic tremor.

No additional neurological abnormalities were reported.

The patient's medical history is notable for bilateral total hip arthroplasty, prior esophagitis, and a history of squamous cell carcinoma.

The clinical presentation is consistent with late-onset focal cervical dystonia with associated dystonic head tremor in the context of a positive family history for similar movement disorders.

Sample 15242 with VPS4A variant:

The patient has a history of dyskinetic cerebral palsy with associated cervical dystonia, with symptom onset occurring in the postnatal period. The clinical course is chronic and non-progressive in terms of the underlying neurological condition, with superimposed movement disorder manifestations.

The patient underwent bilateral C3–C7 instrumented cervical spondylodesis with osteoligamentous decompression at the age of 41 years. Additional relevant diagnoses include cervical myelopathy, as well as prior hardware complications, including left-sided C3 screw dislocation and right-sided C7 rod dislocation.

At age 42, neurological examination demonstrated a clinical picture consistent with dyskinetic cerebral palsy. Superimposed on this baseline condition, the patient exhibited intermittent dystonic cervical movements characterized by jerky torticollis with up to 30° leftward deviation and intermittent retrocollis of approximately 30–40°.

Marked hypertrophy of the bilateral sternocleidomastoid muscles was observed. Gait was spastic in nature, accompanied by a truncal postural deformity.

No cerebral imaging studies were available for review.

Symptomatic management is currently being performed with botulinum toxin injections, targeting cervical dystonia with partial symptomatic benefit.

The clinical presentation is consistent with dyskinetic cerebral palsy with superimposed cervical dystonia, manifesting as intermittent dystonic cervical posturing and associated musculoskeletal complications in the context of long-standing neurodevelopmental motor impairment.
